# Does the Information Quality of ChatGPT Meet the Requirements of Orthopedics and Trauma Surgery?

**DOI:** 10.7759/cureus.60318

**Published:** 2024-05-15

**Authors:** Adnan Kasapovic, Thaer Ali, Mari Babasiz, Jessica Bojko, Martin Gathen, Robert Kaczmarczyk, Jonas Roos

**Affiliations:** 1 Department of Orthopedics and Trauma Surgery, University Hospital of Bonn, Bonn, DEU; 2 Department of Dermatology and Allergy, School of Medicine, Technical University of Munich, Munich, DEU

**Keywords:** large language model, medical information quality, patient education, chatgpt, artificial intelligence in medicine

## Abstract

Background: The integration of artificial intelligence (AI) in medicine, particularly through AI-based language models like ChatGPT, offers a promising avenue for enhancing patient education and healthcare delivery. This study aims to evaluate the quality of medical information provided by Chat Generative Pre-trained Transformer (ChatGPT) regarding common orthopedic and trauma surgical procedures, assess its limitations, and explore its potential as a supplementary source for patient education.

Methods: Using the GPT-3.5-Turbo version of ChatGPT, simulated patient information was generated for 20 orthopedic and trauma surgical procedures. The study utilized standardized information forms as a reference for evaluating ChatGPT's responses. The accuracy and quality of the provided information were assessed using a modified DISCERN instrument, and a global medical assessment was conducted to categorize the information's usefulness and reliability.

Results: ChatGPT mentioned an average of 47% of relevant keywords across procedures, with a variance in the mention rate between 30.5% and 68.6%. The average modified DISCERN (mDISCERN) score was 2.4 out of 5, indicating a moderate to low quality of information. None of the ChatGPT-generated fact sheets were rated as "very useful," with 45% deemed "somewhat useful," 35% "not useful," and 20% classified as "dangerous." A positive correlation was found between higher mDISCERN scores and better physician ratings, suggesting that information quality directly impacts perceived utility.

Conclusion: While AI-based language models like ChatGPT hold significant promise for medical education and patient care, the current quality of information provided in the field of orthopedics and trauma surgery is suboptimal. Further development and refinement of AI sources and algorithms are necessary to improve the accuracy and reliability of medical information. This study underscores the need for ongoing research and development in AI applications in healthcare, emphasizing the critical role of accurate, high-quality information in patient education and informed consent processes.

## Introduction

Artificial intelligence (AI) has been the subject of much interest and research in recent years due to its ability to analyze large amounts of data and recognize patterns that may not be obvious to human experts [1-3]. The concept of AI was first introduced in the 1950s [4]. The main principles are based on the claim that computers could accurately mimic human cognitive functions such as learning and problem-solving [5,6]. Machine learning, a form of AI that uses computerized algorithms that learn and improve with experience, will find increasing application in medicine in the coming years [7]. The AI concepts of deep learning and artificial neural networks have become the cornerstones of significant achievements in image processing [8-10]. Several studies have demonstrated the effectiveness of deep learning algorithms for the interpretation of radiologic images compared to human experts [11-13]. Today, AI is seen as a digital industry that implements new concepts and solutions to tackle complex challenges. With continuous progress in electronic speed, capacity, and software programming, computers are increasingly succeeding in mapping or emulating human intelligence. In the field of medicine, AI has the potential to change the way healthcare is delivered. There is a lack of data in the literature, and it remains unclear whether AI can lead to more accurate diagnoses, better treatment plans, and improved patient outcomes.

A recent example of AI in medicine is Chat Generative Pre-trained Transformer (ChatGPT), a large-scale language model developed by OpenAI that is capable of understanding and generating human-like speech [14]. It was made available to the public in November 2022. ChatGPT has been trained on a huge corpus of text data, including medical literature and patient records, and can answer questions, provide explanations, and generate responses.

The use of ChatGPT in medicine has not been studied in depth. Nevertheless, it could have many potential applications, such as in clinical decision-making, patient education, and disease monitoring [15,16]. In a previous study, a comparative content analysis of health-related information from ChatGPT and selected websites was conducted. Comparable results were obtained with regard to DISCERN ratings [17]. This provides further indications of usability in the future.

Studies are needed to prove whether ChatGPT could help clinicians diagnose patients quickly and accurately by analyzing their symptoms and medical history and suggesting appropriate tests and treatments. Furthermore, it remains unclear whether it can also provide patients with personalized information about their conditions and treatments, helping them to make more informed decisions about their health [18]. ChatGPT could even enable physicians to improve the quality of discharge summaries by inputting specific information to be included [19].

Initial studies compared the knowledge and interpretation ability of ChatGPT with medical students, with ChatGPT's performance being lower than that of medical students, but still good enough to achieve the equivalent of a passing score on a medical exam [20,21]. Other initial investigations of ChatGPT in medical education have found inaccurate and unreliable output data [22].

This study was conducted to understand the role of AI-based language models as a source of information for patients. The aim is to assess the current quality of information, identify limitations in its use, and ultimately determine the potential of large-scale language models as an additional source of information for patient education.

## Materials and methods

The text-based dialog system ChatGPT (OpenAI, San Francisco, CA, USA) was used to simulate information for patients about 20 common orthopedic and trauma surgical procedures. We used 10 primary trauma surgery and 10 primary orthopaedic information forms that are regularly used in the clinic where the study was conducted. For this study, we used the GPT-3.5-Turbo version, which was released on February 13, 2023. 

Study procedures

The following surgical procedures were simulated, and sourced from standardized information forms (Thieme proCompliance) regularly used in the clinical setting (Table 1).

**Table 1:** Investigated surgical procedures

No.	Surgical procedure
1	Stabilization surgery of the degenerative spine
2	Reconstruction of the anterior cruciate ligament (ACL)
3	Total knee replacement (knee arthroplasty)
4	Shoulder joint replacement (shoulder arthroplasty)
5	Revision of total hip replacement
6	Arthroscopy of the knee joint
7	Arthroscopy of the shoulder joint
8	Revision of total knee replacement
9	Total hip replacement (hip arthroplasty)
10	Operations for hallux valgus
11	Operations for lumbar spinal stenosis
12	Stabilization surgery for fracture, inflammation, and tumor
13	Herniated disc surgery
14	Kyphoplasty
15	Removal of osteosynthesis material
16	Joint-preserving surgery for femoral neck fracture
17	Osteosynthesis in adults
18	Osteosynthesis for ankle injuries
19	Osteosynthesis in the arm
20	Osteosynthesis for radius fractures

Relevant keywords were defined by two orthopedic surgeons (JR & AK) from standardized information forms (Thieme proCompliance) for the surgical procedures mentioned. Both the already marked keywords of the information forms and accompanying words relevant to the authors were used. These forms served as a reference for evaluating the answers provided by ChatGPT. The chatbot received identical questions to those in the reference form and provided AI-based answers. The questions were selected based on individual sections of the Thieme forms. Both general questions from the chapters were used, such as "Why is the metal removed/replaced?", as well as specific subcategories that are not formulated as questions in the forms, such as "What are special risks after metal replacement?". The study analyzed the accuracy and quality of the information provided by ChatGPT by comparing the system's responses with the relevant keywords previously defined in the reference forms.

The DISCERN instrument, a validated tool originally consisting of 16 questions, was used for objective information assessment [23]. It measures the accuracy, reliability, and quality of the information presented to patients. As the information sources of ChatGPT remain unknown, questions on information sources could not be applied in this study. Therefore, a modified DISCERN (mDISCERN) instrument with 10 questions was adopted (Table 2).

**Table 2:** mDISCERN assessment questions mDISCERN: modified DISCERN

No.	Question
1	Are the objectives clear and achieved?
2	Is the information presented balanced and unbiased?
3	Are additional sources of information listed as references for patients?
4	Is the mode of action of each treatment described?
5	Are the benefits of each treatment described?
6	Are the risks of each treatment described?
7	Is there a description of how the treatments may affect quality of life?
8	Is it clearly stated that there may be more than one possible treatment?
9	Is the information helpful for "shared decision-making"?
10	Based on the answers to all the previous questions, evaluate the information in terms of its overall quality as a source of information.

However, it must be emphasized that the mDISCERN instrument used in this study is not a validated measurement instrument, as it differs from its original form. The calculation of the mDISCERN score is analogous to the original variant. A higher value in the mDISCERN means greater reliability. If an mDISCERN score of 3 or higher was achieved, the information was classified as very reliable. As in the original study, between 1 and 5 points could be awarded for each question.

In addition, a global medical assessment of the information provided by ChatGPT was also carried out. The following categories were distinguished: "very useful," "somewhat useful," "not useful," and "dangerous."

Statistical analysis was performed using Python v3.11.2 (Python Software Foundation) and the statistical libraries SciPy v1.10.1 (The SciPy Community) and Pandas v1.5.3 (Python Software Foundation). Pearson correlation coefficients were calculated to evaluate the linear correlation between the given variables. The diagrams were created with the Python libraries Matplotlib v3.7.0 and Seaborn v0.12.2.

## Results

**Figure 1:**
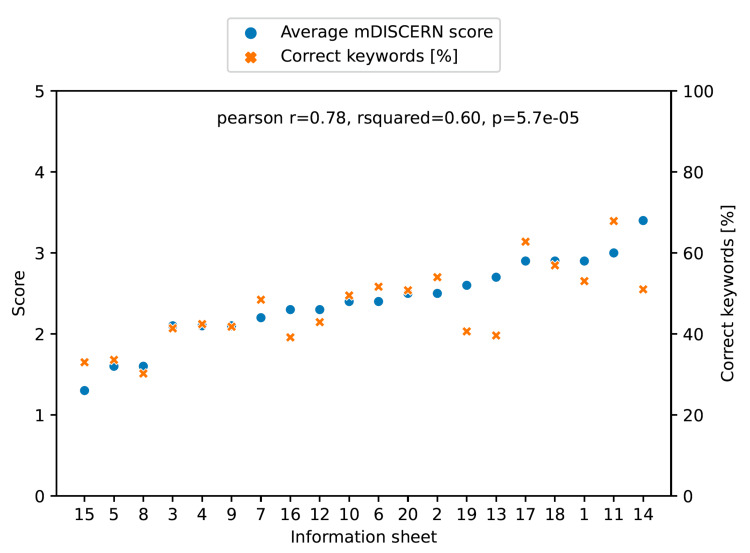
Correlation of keywords correctly identified by ChatGPT (orange x) and the average mDISCERN score (blue dot) The order of the information sheet is shown in Table 1 mDISCERN: modified DISCERN

The number of relevant keywords in the referenced informed consent forms varied between 32 and 90. On average, 47% of relevant keywords were mentioned by ChatGPT (Figure 1; lowest: 30.5% for revision total knee replacement, highest: 68.6% for surgery for lumbar spinal stenosis). The average mDISCERN score was 2.4 out of 5 points (see Figure 1; lowest: 1.3 for removal of osteosynthesis material, highest: 3.4 for kyphoplasty).

**Figure 2:**
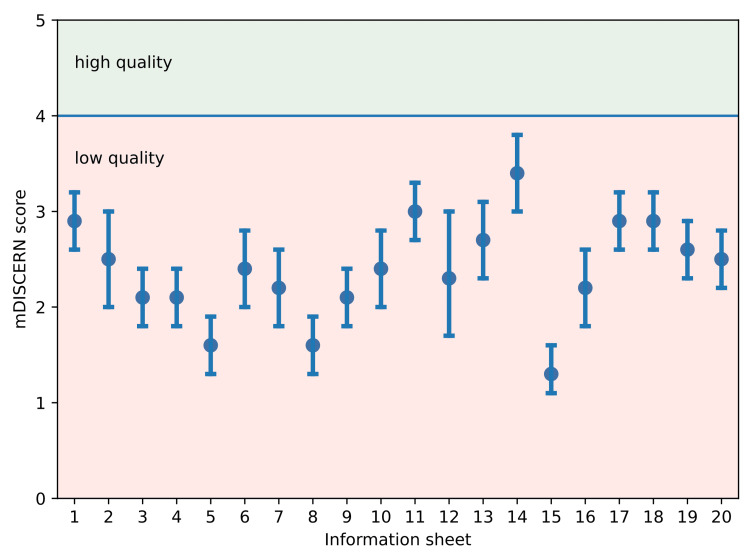
Average mDISCERN score per information sheet (with 95% confidence interval); information was categorized as high quality if the mDISCERN score was 4 or higher mDISCERN: modified DISCERN

Based on responses to all previous questions, no total Chat-GPT information was rated as "high - minimal deficiencies" (average mDISCERN score 4 or higher), while nine (45%) cases were rated as "moderate - potentially important but not significant deficiencies" and 11 (55%) cases were rated as "low - significant deficiencies" (Figure 2).

**Figure 3:**
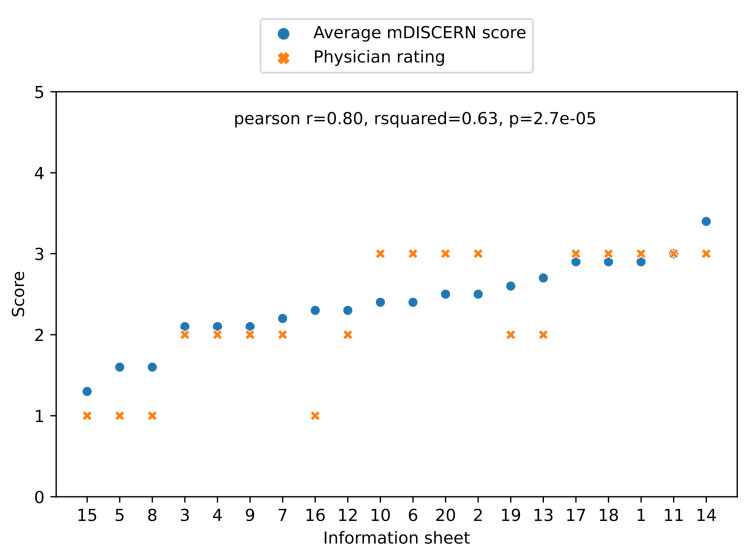
Correlation of the average mDISCERN score with the global rating by physicians (4 = "very useful," 3 = "somewhat useful," 2 = "not useful," and 1 = "dangerous") mDISCERN: modified DISCERN

Of the 20 fact sheets provided by ChatGPT, none were rated as medically sound or "very useful," nine (45%) were rated as "somewhat useful," seven (35%) were rated as "not useful," and four (20%) were considered misleading or "dangerous" in a global physician assessment (Figure 3). There was a positive correlation between a higher mDISCERN score and a better physician rating (Figure 3), meaning that information with a lower mDISCERN score was more likely to be considered "not useful" or "dangerous."

## Discussion

The integration of AI-based systems like ChatGPT into patient education and medical processes offers significant promise for advancements in healthcare. However, discrepancies have been identified, particularly in a study comparing ChatGPT with Google web search as health information sources, especially in response to common questions about knee and hip arthroplasty. This study found that only a quarter of the responses were similar between the two sources, with ChatGPT providing varied answers that underscore its reliability issues [24,25]. Our research corroborates this finding, highlighting the need for improvement in the accuracy of medical information. Nonetheless, these models are under continuous development, stressing the importance of further research and comparison of different language models in future studies. AI-based chatbots, by delivering precise, reliable, and comprehensible information regarding surgical procedures, hold the potential to significantly enhance patient satisfaction. Additionally, the future development of procedure-specific graphics could further elevate patient education. Using AI systems can also streamline the education process and informed consent, saving valuable time and resources.

Despite the promising benefits of AI in medicine, challenges and limitations exist. For instance, our findings indicate that the current version of ChatGPT, 3.5-Turbo, falls short of meeting medical information standards in orthopedics and traumatology. Another study showcased a chatbot named Felix that improved the postoperative experience for hip arthroscopy patients by efficiently addressing 79% of inquiries, although it failed to recognize or address health concerns in some cases [26]. This emphasizes the critical need for accurate responses and highlights the risks associated with providing incorrect or incomplete information through chatbots.

The provision of medical information during the education for surgical procedures is crucial in the doctor-patient relationship and requires high-quality medical information. For example, a previous study showed that videos about elbow fractures can convey medical content and thus serve as a source of information [27]. Yet, the information quality in our study was found to be average to low when compared to standard information sheets in regular use, with some being overly general. Further research is needed to assess the quality of information for specific interventions. Additionally, with 93% of ChatGPT's training on English datasets, the impact of language on the quality of information warrants further examination [28].

Given that ChatGPT produces answers that seem realistic and intelligent, it's imperative that these responses are meticulously verified. The anonymity of ChatGPT's sources presents a security concern, as the origins and quality of the medical information provided cannot be assessed. The answers provided may contain both true and fictitious information. Continuous research is therefore necessary in order to utilize the potential in the future and to know the limits [29]. Despite these limitations, ChatGPT has various potential applications in orthopedics, ranging from streamlining paperwork to aiding in diagnosis, surgical planning, and research [30].

In our study, 20% of the information provided by ChatGPT was classified as dangerous, with the potential to mislead patients. This raises major concerns about ethical and legal implications in the use of AI in healthcare, including issues of privacy, liability, and bias. However, with the ability to analyze vast amounts of data, recognize patterns, and generate insights, AI has the ability to improve decision-making, increase surgical precision, and ultimately shape the future of orthopedic surgery [31]. Nevertheless, there appears to be a discrepancy in terms of applicability and actual use. In particular, the uninformed user runs the risk of falling for false or inadequate information, whereas the informed user has the potential to use it to the full.

In addition, there may be concerns that AI could replace human expertise and decision-making, which could lead to a loss of empathy and compassion in patient care. Nevertheless, the quality of medical information provided by ChatGPT in our study is currently insufficient to provide adequate information quality for medical intervention. There is currently a need for regulators and healthcare professionals to develop standards for minimum quality and to raise patient awareness of the current limitations of AI assistants. The selection of keywords used was ultimately based only on subjective criteria and pre-labeled terms and was only made by two physicians in this study. In the future, this selection should be validated both by a larger group of professionals and by obtaining patient evaluations in order to check the quality of the information and compare it with the quality of medical information. This is the aim of future studies.

Limitations

This study has several limitations that should be considered. First, the mDISCERN instrument used to assess the quality of medical information provided by ChatGPT has not been validated, so its results may not accurately reflect the reliability or quality of the information assessed. In addition, the study relied on a limited number of surgical procedures, and the keywords used for the assessment were selected by only two orthopedic surgeons, which may introduce bias and limit the generalizability of the results. Another important limitation is that the sources of information used by ChatGPT could not be verified, which casts doubt on the accuracy and reliability of the content generated by ChatGPT.

## Conclusions

In summary, the use of AI-based systems such as ChatGPT in medicine has the potential to greatly improve patient education and informed consent processes in the future. However, the quality of medical information in the field of orthopedics and trauma surgery currently provided by ChatGPT is low and not comparable to a physician. The authors point out that it remains unclear which sources the AI system draws on for a particular answer. Further research is needed to determine whether the quality of medical information provided by ChatGPT can be significantly improved through future software and source enhancements.
